# Acral lentiginous melanoma demonstrates more aggressive pathological features than non-acral melanoma in Jamaica: A 10-year multicenter study

**DOI:** 10.1016/j.jdin.2025.06.005

**Published:** 2025-07-10

**Authors:** Chico J. Collie, Simone L. Jean-Marie, Rory K. Thompson, Nadia P. Williams, Jonathan D. Ho

**Affiliations:** a Department of Pathology, The University of the West Indies, Mona Campus, Jamaica, West Indies; b Division of Dermatology, Department of Medicine, The University of the West Indies, Mona Campus, Jamaica, West Indies

**Keywords:** Afro-Caribbean, Breslow, Jamaica, melanoma, prognosis, skin of color, epidemiology

*To the Editor:* Acral lentiginous melanoma (ALM) presents late with worse pathologic prognostic features and clinical outcomes compared with nonacral subtypes.^1^ Pathologic characteristics of the primary tumor, particularly depth and ulceration, are vitally important prognostic predictors. We examine the pathological prognostic features of cutaneous melanoma in Jamaica, a predominantly Afro-Caribbean population, and compare features of acral and nonacral subtypes.

We reviewed histopathology reports of cutaneous melanoma (from January 2013 to September 2023) at The University of the West Indies, Dermatopathology Laboratory Services, and Diagnostic Pathology Services. Demographics, clinical features, subtypes, mandatory reporting, histopathologic features, and nodal evaluation/status were recorded. Invasive melanomas were categorized into thin (≤1.0 mm), intermediate (1.1-4.0 mm), thick (4.1-8 mm), and ultrathick (>8 mm). Descriptive/comparative statistics were generated/analyzed (χ^2^/t tests; IBM SPSS V26).

We identified 125 cases (119 patients). The mean age was 65.9 (±13.5) years. The male-to-female ratio = 1.1:1. ALM (60.8%, *n* = 76) predominated over nonacral melanoma (NAM, 39.2% [*n* = 49]). ALMs were significantly thicker than NAM (mean = 7.2 mm vs 3.1 mm, *P =* .001) and were significantly more likely to be thick/ultrathick (67.2% vs 13.5%, *P =* .001). T-stages are shown in Figure 1. ALM was more commonly ulcerated (68.3% vs 21.6%, *P =* .001) with higher mitotic rates (mean difference 1.9/mm^2^, *P =* .011) and suspected or present lymphovascular invasion (LVI, *P =* .048). Regression was more common in NAM (8.8% vs 0%, *P =* .022*).* See Table I. No significant differences were noted for microsatellites, tumor-infiltrating lymphocytes, or neurotropism. Sentinel lymph node biopsy was indicated in 55.2% but performed in only 1/3. No difference in nodal involvement (*P =* .66) was found. Ultrathick melanomas were more likely to have nodal disease than thick melanomas (*P =* .024).

**Fig 1:**
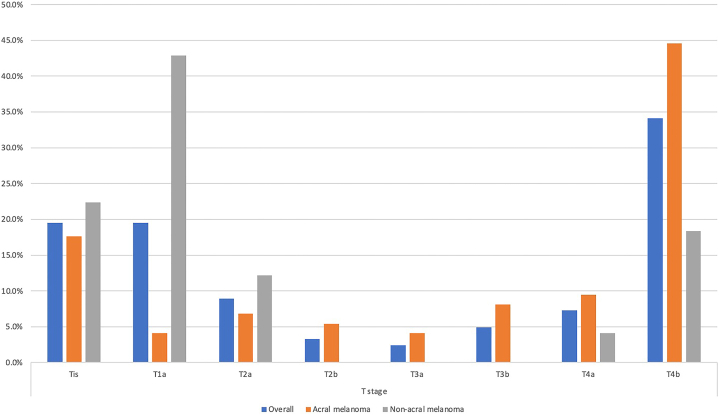
T stage for acral and nonacral melanoma in a Jamaican population. Our data show high proportions of thick and ulcerated melanomas (T4b) in acral lentiginous melanomas compared with nonacral subtypes (odds ratio 3.6; 95% CI, 1.5-8.4, *P* = .0035). Similarly, thin, nonulcerated lesions (T1a) were markedly more common in nonacral lesions compared with acral melanoma (odds ratio 17.8; 95% CI, 4.9-64.2, *P* < .0001).

**Table I:** Pertinent histological features

Histological parameter	Overall	ALM	NAM	*P*
Ulceration (available, *n* = 100)	51% (*n* = 51)	68.3% (*n* = 43)	21.6% (*n* = 8)	.001∗
Mean thickness (available, *n* = 98)	5.7 mm	7.2 mm (*n* = 61, SD ± 6.0)	3.1 mm (*n* =37, SD ± 4.9)	MD: 4.1 mm .001∗
Thickness category (*n* = 98)				
Thin ≤ 1.0 mm	23.5% (*n* = 23)	4.9% (*n* = 3)	54.1% (*n* =20)	.001∗
Intermediate 1.1-4.0 mm	25.5% (*n* = 25)	27.9% (*n* = 17)	21.6% (*n* = 8)	.47
Thick 4.1-8 mm	25.5% (*n* = 25)	32.8% (*n* = 20)	13.5% (*n* = 5)	.001∗†
Ultrathick > 8 mm	25.5% (*n* = 25)	34.4% (*n* = 21)	10.8% (*n* = 4)	.001∗†
Mean mitotic rate (per mm^2^) (available, *n* = 100)	2.4	3.1 (*n* = 63, SD ± 4.1)	1.2 (*n* = 37, SD ± 2.6)	MD: 1.9 .011∗
Lymphovascular invasion (available, *n* = 100)	18% (*n* = 18)	23.8% (*n* = 15)	8.1% (*n* = 3)	.048∗
Tumor regression (available, *n* = 100)	3.0% (*n* = 3)	0.0% (*n* = 0)	8.8% (*n* = 3)	.022∗
Tumor-infiltrating lymphocytes (available, *n* = 100)	30% (*n* = 30)	23.4% (*n* = 15)	40.5% (*n* = 12)	.078∗
Microsatellitosis (available, *n* = 100)	8% (*n* = 8)	11.1% (*n* = 7)	2.7% (*n* = 1)	.135∗
Neurotropism (available, *n* = 100)	6.0% (*n* = 6)	9.5% (*n* = 6)	0.0% (*n* = 0)	.082∗
Overall melanoma data Total no. of cases of melanoma: 125 Acral melanoma: 60.8% (*n* = 76) NAM: 39.2% (*n* = 49) Melanoma by growth phase Invasive melanoma 80.8% (*n* = 101) Melanoma in-situ 19.2% (*n* = 24)		Melanoma by subtype Non-UVR-related ALM 60.8% (*n* = 76) Melanoma arising in congenital nevi 0.8% (*n* = 1) UVR-related Low-CSD melanoma 31.2% (*n* = 39) High-CSD melanoma 6.4% (*n* = 8) Desmoplastic melanoma 0.8% (*n* = 1)		

ALMs were more likely than nonacral melanomas to have aggressive pathologic features, such as thick/ultrathick depths, ulceration, increased mitotic activity, and lymphovascular invasion. Regression, a potentially protective feature was significantly less common in ALM.

*ALM*, Acral lentiginous melanoma; *CSD*, cumulative sun damage; *MD*, mean difference; *NAM*, nonacral melanoma; *UVR*, UV radiation.

∗ Significant values (*P* < .05).

† Evaluated together as thick or ultrathick ie, pT4 stage.

ALM predominates over NAM in our population. The data highlight aggressive pathologic features and potentially worse predicted outcomes. This is best demonstrated by mean depth and ulceration status. Prognostically, T1a-stage tumors (most frequently occurring T stage in NAM cases), have reported 5 or 10-year melanoma-specific survival rates of 99%/98% compared with 82%/75% in T4b tumors (most frequently occurring T stage in our ALM patients).^2^ Other aggressive features, including LVI and elevated mitotic rates, were also more common in ALM. Contrastingly, regression, reportedly associated with improved outcomes, was significantly more common in NAMs.^3^ Similarity in nodal involvement rates between groups was unexpected, but we suspect this may be due to overall low rates of nodal evaluation in indicated cases.

Advanced stage at presentation is consistently reported for ALM. Delayed diagnosis (late presentation, hidden anatomical sites, and physician misdiagnosis), innate differences in tumor biology, relative lack of health care access in populations commonly affected by ALM, and decreased melanoma awareness are suggested causes.^4^^,^^5^ This combination likely contributes in our setting. Specifically, there is relatively low melanoma awareness in Jamaica.^5^ For the minority with awareness, knowledge centers around UV-related tumors/prevention with poor ALM-specific knowledge. This is concerning given the high proportion of ALM seen in an Afro-Caribbean predominant population. Increased public health efforts aimed at increasing ALM awareness could combat one cause of delayed/advanced presentation.

We report pathologic/prognostic features of ALM in a Jamaican population. Limitations include the retrospective nature, missing data points, lack of longitudinal data, and sampling a primarily urban cohort.

## Conflicts of interest

None disclosed.
